# Case Report: Effects of Secondary Hyperparathyroidism Treatment on Improvement of Juvenile Nephronophthisis-Induced Pancytopenia and Myelofibrosis

**DOI:** 10.3389/fped.2021.550158

**Published:** 2021-05-11

**Authors:** Keishiro Amano, Hidemi Toyoda, Kouhei Nishikawa, Tomohiro Murata, Masahiro Hirayama

**Affiliations:** ^1^Department of Pediatrics, Mie University Graduate School of Medicine, Tsu, Japan; ^2^Department of Nephro-urologic Surgery and Andrology, Mie University Graduate School of Medicine, Tsu, Japan; ^3^Department of Cardiology and Nephrology, Mie University Graduate School of Medicine, Tsu, Japan

**Keywords:** myelofibrosis, pancytopenia, hyperparathyoidism, end-stage-renal disease, nephronophthisis

## Abstract

Secondary hyperparathyroidism (HPT) is a common complication of end-stage renal disease (ESRD) and may be an important precipitating factor for the development of myelofibrosis. However, there have been only a few reports on myelofibrosis caused by secondary HPT in children. We describe a case of a 15-year-old boy with myelofibrosis due to secondary HPT who was successfully treated with hemodialysis, erythropoietin, phosphate binders, and activated vitamin D agents. The patient had no past medical history and had been admitted to the hospital for abdominal pain. Routine blood examination revealed pancytopenia combined with renal impairment. Hyperphosphatemia, decreased 1,25-dehydroxyvitamin D, decreased serum calcium, and increased parathyroid hormone (PTH) levels were observed. Bone marrow biopsy confirmed myelofibrosis and renal biopsy revealed nephronophthisis (NPHP). The possibility of renal osteodystrophy and myelofibrosis due to secondary HPT was considered. Hemodialysis and erythropoietin were initiated and combined therapy with a phosphate binder and an active vitamin D agent achieved greater reduction of PTH levels, along with improvement of pancytopenia. As medical treatment for secondary HPT can lead to a reversal of myelofibrosis and avoid parathyroidectomy in children, prompt recognition of this condition has major implications for treatment. Therefore, despite its rarity, pediatricians should consider myelofibrosis due to secondary HPT as a cause of pancytopenia in patients with chronic kidney disease.

## Introduction

Myelofibrosis can occur *de novo* (primary myelofibrosis) or secondary to a number of pathologic states such as various neoplasms, renal osteodystrophy, infections and collagen diseases (secondary myelofibrosis) (1). Somatic *JAK2 V617F* mutations are found in approximately 50% of adult patients with primary myelofibrosis. An additional 5–10% of patients have a somatic mutation in *MPL* (*MPL W515K/L*), which leave ~40% of primary myelofibrosis patients without an identified mutation (1). However, *JAK2 V617F* and *MPL* mutations are extremely rare in children with primary myelofibrosis. It has been suggested that excess secretion of parathyroid hormone (PTH) stimulates bone marrow fibroblasts, leading to myelofibrosis (2, 3). Consistent with this finding, transgenic mice carrying constitutively active PTH-related peptide receptors in osteoblasts show myelofibrosis (4, 5).

Secondary hyperparathyroidism (HPT) is a common and severe complication of end-stage renal disease (ESRD), and has an unfavorable impact on patients' outcomes, particularly in those undergoing hemodialysis (6). Treatment of HPT in ESRD consists of dietary modification, phosphate binders, and administration of active vitamin D (7). The introduction of targeted therapies, such as selective calcium-sensing receptor (CaSR) modulators, offers more opportunity to adequately control elevated PTH levels, especially in patients with ESRD on dialysis (8). Although parathyroidectomy can yield a dramatic reduction in PTH levels and clinical symptoms, surgery for secondary HPT has significantly higher rates of morbidity and mortality, and require special postoperative attention (9). Therefore, parathyroidectomy should be a last resort and only be performed after evidence of failure of medical therapy, followed by careful evaluation for all potential complications of such surgical procedures (6).

Nephronophthisis (NPHP) is characterized by the reduced ability of the kidneys to concentrate solutes, chronic tubulointerstitial nephritis, cystic renal disease, chronic tubulointerstitial nephritis, and progression to ESRD (10). NPHP is one of the most common genetic disorders causing ESRD in children and adolescents. The typical clinical symptoms of NPHP include polyuria, polydipsia, secondary enuresis, anemia and growth retardation. Three clinical subtypes of NPHP have been recognized based on the median age of onset of ESRD: infantile (onset of ESRD prior to 4 years of age), juvenile (mean age of onset of ESRD of 13 years), and adolescent (onset of ESRD at a mean age of 19 years) (10). NPHP occurs as an isolated kidney disease, but ~15% of patients have extrarenal manifestations of ciliopathy syndromes, such as retinal defects, liver fibrosis, skeletal abnormalities, and brain developmental disorders (11). The pleiotropy of NPHP is explained by the fact that almost all NPHP gene products share their expression in primary cilia, a sensory organelle present in most mammalian cells (11). Although more than 25 different genes have been found to be associated with NPHP, mutations in the *NPHP1* gene are the most common, being reported in ~one-third of affected patients (10). However, the causative mutations of NPHP remain unknown in the remaining two-thirds of cases (11).

Here, we report the case of a boy who presented with myelofibrosis due to secondary HPT, which led to the diagnosis of NPHP. To our knowledge, this is the first report on myelofibrosis resulting from secondary HPT caused by NPHP. Myelofibrosis caused by secondary HPT is uncommon in children. Pediatricians should be aware of this condition, as optimal medical therapy for secondary HPT can lead to a reversal of myelofibrosis.

## Case Report

**Figure 1:**
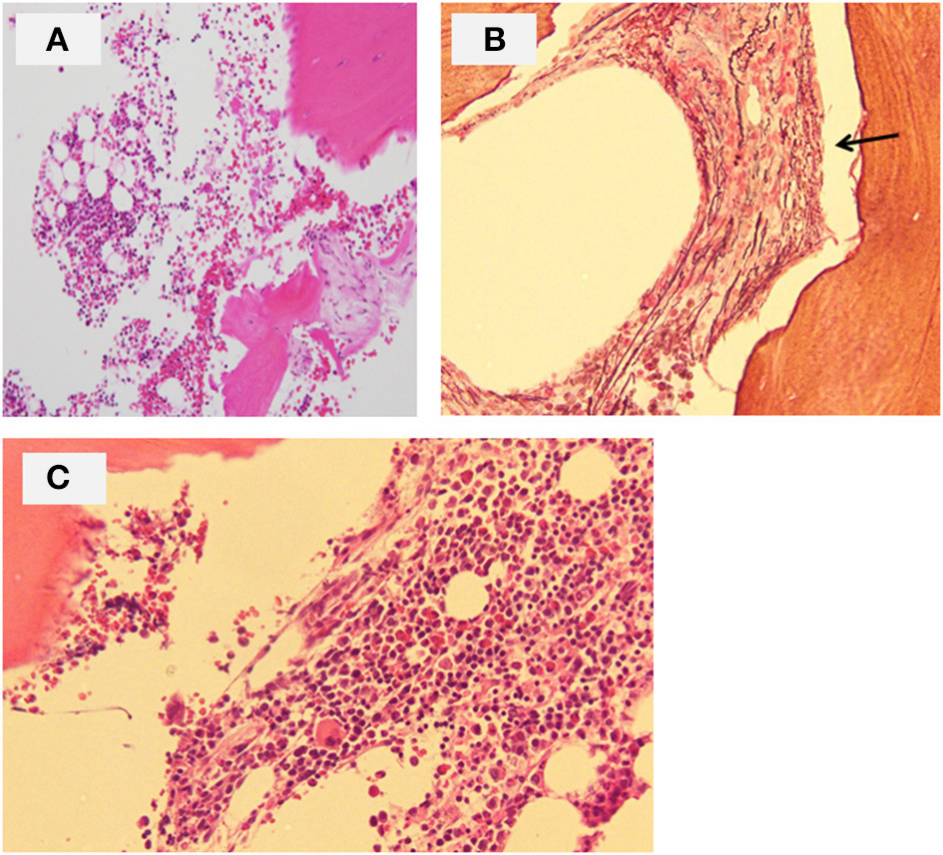
Bone marrow biopsy of the patient. **(A)** Hematoxylin and eosin section at onset showing a hypocellular marrow with trilineage hypoplasia (original magnification ×40). **(B)** Silver staining section at onset showing increased diffuse coarse reticulin (arrow) (original magnification ×100). **(C)** Hematoxylin and eosin section following pharmacological treatment for hyperparathyroidism showing normal trilineage hematopoiesis and complete reversal of fibrosis (original magnification ×100).

**Figure 2:**
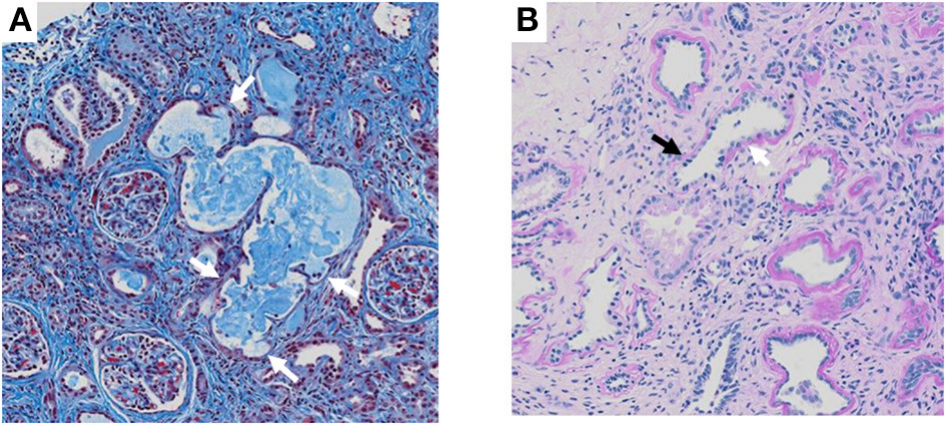
Renal biopsy histopathology. **(A)** Masson staining section showing irregular tubular dilatation with diverticular outpouchings (white arrow). **(B)** Periodic acid silver staining section showing thickening (white arrow) and thinning (black arrow) of tubular basement membrane and chronic interstitial inflammation.

**Figure 3:**
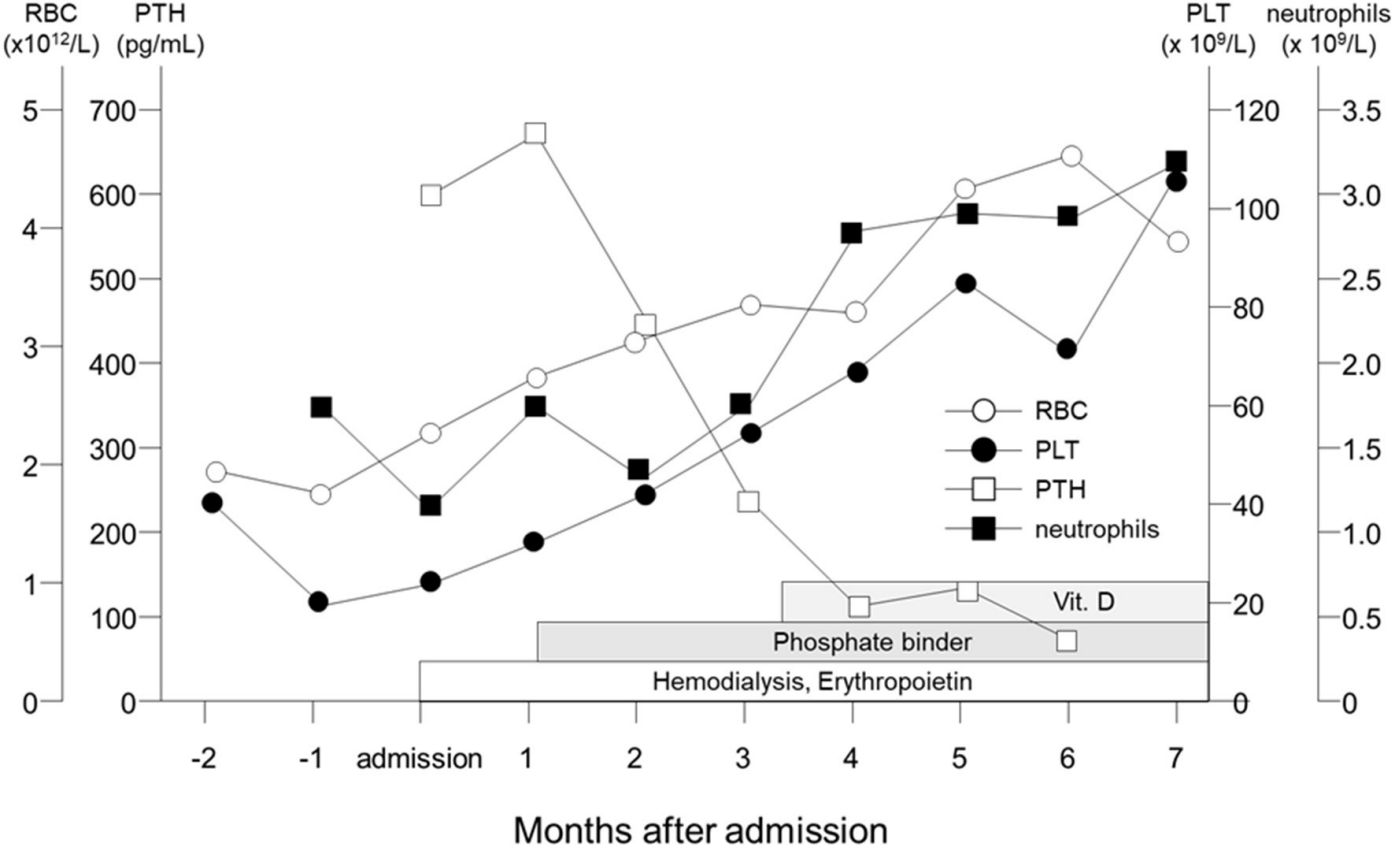
Clinical course of the patient. Parathyroid hormone levels (open square) are shown to fall and neutrophils (filled square), erythrocyte counts (open circle) and platelets (filled circle) are shown to rise gradually following HD and pharmacological therapy with Epo, phosphate binder and active vitamin D.

The patient was the first son of healthy and non-consanguineous Japanese parents, born at term following an uncomplicated pregnancy. There was no family history of renal insufficiency or hematological disorders. His past medical history was unremarkable. At the age of 15 years, he was incidentally noted to have renal impairment combined with pancytopenia while being reviewed for abdominal pain. On examination, he looked pale. He did not have polydipsia/polyuria, growth retardation, hypertension, hepatosplenomegaly, lymphadenopathy or petechiae. The patient's height was 165 cm [−0.5 standard deviation (SD)]. Peripheral blood analysis revealed anemia and thrombocytopenia with an erythrocyte count of 1.98 × 10^12^/L, a hemoglobin (Hb) level of 5.8 g/dl and platelet count of 22 × 10^9^/L. White blood cell and neutrophil counts were 2.6 × 10^9^/L and 1.3 × 10^9^/L, respectively. Laboratory investigations revealed renal impairment with blood urea nitrogen (BUN) of 150 mg/dl and creatinine (Cr) of 9.12 mg/dl. Hyperphosphatemia (9.6 mg/dl) and decreased serum calcium (5.4 mg/dl) were observed. Apart from mild proteinuria, urine analysis detected no pathological findings. Other laboratory parameters were normal except for hyperuricemia (13.2 mg/dl). Ultrasonography revealed enlarged parathyroid glands, normal-sized kidneys, and multiple bilateral renal cysts with loss of corticomedullary differentiation. A bone marrow biopsy revealed a hypocellular marrow with trilineage hypoplasia (Figure 1A). Increased bone remodeling with fibrosis and increased diffuse coarse reticulin was confirmed by silver staining (Figure 1B). There was no evidence of metastatic tumors or myeloproliferative neoplasms. Molecular studies looking for *JAK2 V617F* and *MPL* mutations, and *BCR/ABL1* rearrangement were normal. His renal biopsy specimen was suggestive of NPHP (Figure 2). Several tubules showed irregular tubular profiles with diverticular outpouchings (Figure 2A). Furthermore, thickening and thinning of the tubular basement membrane and chronic interstitial inflammation were observed (Figure 2B). The PTH level was significantly increased (585 pg/ml; normal range, 15-65 pg/ml). The overall findings were diagnostic of non-neoplastic myelofibrosis resulting from secondary HPT in a patient with ESRD caused by NPHP. Hemodialysis (HD) and erythropoietin (dosage 75 U/kg/dose × 3 times/week) were initiated, and combined therapy with a phosphate binder and an active vitamin D agent achieved reduction of PTH levels, along with improvement of pancytopenia (Figure 3). Bone marrow biopsy showed normal trilineage hematopoiesis and a complete reversal of fibrosis (Figure 1C). The patient remained on HD for 3 years and underwent successful deceased-donor renal transplantation at 18 years of age.

## Discussion

Myelofibrosis resulting from secondary HPT caused by ESRD is an uncommon disease in young individuals (aged < 20 years old), and only few cases have been reported (Table 1) (12–14). Treatment of HPT in renal disease consists of dietary modification, phosphate binders, and active vitamin D (7). When medical treatment fails or is not tolerated because of adverse effects in patients with severe secondary HPT, surgical parathyroidectomy is the final therapeutic option, and is required in about 5% of patients (15, 16). Parathyroidectomy ameliorates anemia and reduces the requirement of postoperative erythropoiesis-stimulating agents (17). There have been reports of patients with myelofibrosis caused by secondary HPT, who were successfully treated using a parathyroidectomy (13, 14, 18). However, cinacalcet has been effective even in patients with marked parathyroid hyperplasia, suggesting that it may serve as an alternative to parathyroidectomy for treatment of severe secondary HPT (15). Furthermore, it has been suggested that cinacalcet improves anemia in chronic hemodialysis patients with secondary HPT (17). Since our patient's pancytopenia and secondary HPT were successfully managed by HD, erythropoietin, phosphate binders and activated vitamin D agents, he did not need parathyroidectomy or cinacalcet.

**Table 1:** Myelofibrosis in pediatric cases (aged <20 years old) with end-stage renal disease in the literature.

**Age Gender**	**Hb (g/dl)**	**Platelet (×10^**9**^/L)**	**BUN (mg/dl)**	**Cr (mg/dl)**	**PTH (pg/ml)**	**Underlying disease**	**Treatment**	**References**
9 yr F	4.3	6	216	17.5	1600	Renal dysplasia	Vit.D, HD	(12)
10 yr F	6.5	1.7	47	2.7	3100	Polycystic kidney	Vit.D, Cinacalcet, Parathyroidectomy	(13)
2 yr M	5.8	0.8	91	0.9	2500	Interstitial nephritis	Cinacalcet, Phosphate binder	(13)
17 yr M	5.4	57			3027	Undetermined etiology	Parathyroidectomy	(14)
15 yr M	5.8	2.6	150	9.12	585	NPHP	HD, EPO, Vit. D Phosphate binder	Our case

Ultrasonography is useful for predicting the response of secondary HPT to medical treatment and to assess the regression of parathyroid gland hyperplasia by measuring parathyroid gland volume (19). When nodular hyperplasia of the parathyroid gland develops, medical therapy for secondary HPT is more likely to fail, and surgery may be indicated (19). It was also reported that patients with at least one parathyroid gland with volume >0.5 cm^3^ (>1 cm in diameter) were usually refractory to pharmacological therapy (20). Since the parathyroid glands of our patient showed diffuse hyperplasia and was <1 cm in diameter (data not shown), this might be a reason for successful pharmacological treatment for secondary HPT. Furthermore, the PTH level was relatively lower (585 pg/ml) in our patient than in those patients who needed parathyroidectomy (≥3000 pg/ml) (Table 1) (13, 14). PTH levels may also potentially identify patients who will benefit from pharmacological treatment.

Myelofibrosis is associated with a number of pathological conditions. While recent investigations have identified important constituents within the fibrotic marrow in patients with certain hematologic malignancies, little is known about the effect of PTH-induced myelofibrosis. Myelofibrosis improves, and serum erythropoietin and blood reticulocytes increase after parathyroidectomy in patients with secondary HPT caused by ESRD (2, 3, 21). Furthermore, primary HPT can also induce myelofibrosis (21). Therefore, excess secretion of PTH leads to myelofibrosis, but uremic toxins or other factors associated with ESRD are less likely to cause it. It has also been reported that intense staining for IL-1a, IL-6, IL-11, TNF-α, and TGF-β was evident within the fibrotic tissues of the bone marrow in dialysis patients with secondary HPT (22). This suggests that selective cytokine accumulation might play a role in modulating bone marrow cell function in ESRD. Although the PTH concentration in our patient was not too high, marked hypocellularity and extensive myelofibrosis were observed. The high sensitivity of the bone marrow to PTH may be related to the classical association of NPHP with anemia.

Important determinant factors for the height of patients with ESRD are the etiology of the disease, age of onset, and duration of CKD (23). Our patient had normal growth despite chronic and advanced renal failure. At the age of 10 years, his height was 135 cm (-0.45 SD). A hematologic workup showed no cytopenia, with a Hb concentration of 11.7 g/dl and platelet count of 167 × 10^9^/L. Laboratory investigations revealed normal renal function with BUN 8.0 and Cr 0.32 mg/dl. This means that the age of CKD onset was at most 10 years or older, and the duration of CKD was 5 years at most. Late onset and short duration of CKD contributed to normal growth in our patient. The diagnosis of NPHP is made by renal biopsy or genetic analysis (11). However, the role of renal biopsy in the diagnosis of NPHP is controversial, and a positive genetic test is required to confirm the diagnosis (10, 11). The genetic test was not performed in our case because of patient refusal. Our patient did not have any extrarenal phenotype associated with NPHP, and the renal biopsy specimen was suggestive of NPHP. However, molecular genetic analysis should be considered in our patient because it is currently the only method for the definitive diagnosis of NPHP.

Myelofibrosis resulting from HPT is uncommon in children. Our case highlights the importance of being aware of myelofibrosis as a potential complication of secondary HPT. Pediatricians should consider this condition when faced with progressive pancytopenia in patients with ESRD because pharmacological treatment can lead to a reversal of myelofibrosis and successful renal transplantation.

## Data Availability Statement

The original contributions presented in the study are included in the article, further inquires can be directed to the corresponding author/s.

## Ethics Statement

Written informed consent was obtained from the individual(s), and minor(s)' legal guardian/next of kin, for the publication of any potentially identifiable images or data included in this article.

## Author Contributions

KA, HT, and MH conceptualized and designed the study, drafted the initial manuscript, and reviewed and revised the manuscript. KN and TM collected data, carried out the initial analyses, and reviewed and revised the manuscript. All authors contributed to the article and approved the submitted version.

## Conflict of Interest

The authors declare that the research was conducted in the absence of any commercial or financial relationships that could be construed as a potential conflict of interest.
